# Mindfulness training increases cooperative decision making in economic exchanges: Evidence from fMRI

**DOI:** 10.1016/j.neuroimage.2016.05.075

**Published:** 2016-09

**Authors:** Ulrich Kirk, Xiaosi Gu, Carla Sharp, Andreas Hula, Peter Fonagy, P. Read Montague

**Affiliations:** a Department of Psychology, University of Southern Denmark, 5230 Odense, Denmark; b Center for Brain Health, School of Behavioral and Brain Sciences, The University of Texas at Dallas, Dallas, TX 75235, USA; c Department of Psychology, University of Houston, Houston, TX 77004, USA; d Wellcome Trust Centre for Neuroimaging, University College London, London WC1N 3BG, UK; e Research Department of Clinical, Educational, and Health Psychology, University College London, London WC1E 6BT, UK; f Anna Freud Centre, London NW3 5SD, UK; g Human Neuroimaging Laboratory, Virginia Tech Carilion Research Institute, Roanoke, VA 24016, UK

**Keywords:** Decision making, fMRI, Social cooperation, Interoception, Mindfulness, Ultimatum Game

## Abstract

Emotions have been shown to exert influences on decision making during economic exchanges. Here we investigate the underlying neural mechanisms of a training regimen which is hypothesized to promote emotional awareness, specifically mindfulness training (MT). We test the hypothesis that MT increases cooperative economic decision making using fMRI in a randomized longitudinal design involving 8 weeks of either MT or active control training (CT). We find that MT results in an increased willingness to cooperate indexed by higher acceptance rates to unfair monetary offers in the Ultimatum Game. While controlling for acceptance rates of monetary offers between intervention groups, subjects in the MT and CT groups show differential brain activation patterns. Specifically, a subset of more cooperative MT subjects displays increased activation in the septal region, an area linked to social attachment, which may drive the increased willingness to express cooperative behavior in the MT cohort. Furthermore, MT resulted in attenuated activity in anterior insula compared with the CT group in response to unfair monetary offers post-training, which may suggest that MT enables greater ability to effectively regulate the anterior insula and thereby promotes social cooperation. Finally, functional connectivity analyses show a coupling between the septal region and posterior insula in the MT group, suggesting an integration of interoceptive inputs. Together, these results highlight that MT may be employed in contexts where emotional regulation is required to promote social cooperation.

## Introduction

Human altruism and social attachment have evolved to promote cooperation and prosocial behavior beyond bonds of kinship (Fehr and Fischbacher, 2003, Trivers, 1971). The ability to regulate emotional reactions in economic and social exchanges is a fundamental component of cooperative behavior among humans. The core question addressed in the present study is whether psychological training, specifically mindfulness training (MT), may enhance cooperative economic decision making. MT cultivates nonjudgmental observation of thoughts and emotions (Kabat-Zinn, 1990), and it remains unexplored whether practices of non-identification with arising states of mind may empower people to change behavior (such as economic decision making) as opposed to reinforcing automatic and habitual responding. In the nascent research field of probing behavioral and neural effects of MT, an important next question to address is whether MT allows individuals to maintain cooperative behavior when confronted with negative emotions. In other words, we ask the question whether individuals are able to inhibit reactions to negative emotions, such that a social exchange may maximize collective earnings. Indeed, recent reports suggest that compassion meditation decrease altruist punishment (McCall et al., 2009) and increase altruistic helping behavior (Weng et al., 2015).

In the current longitudinal randomized controlled fMRI study using the Ultimatum Game, we compare a subject cohort who received 8 weeks of MT to a subject cohort who received 8 weeks of active control training (CT) in the form of physical relaxation training. Both interventions focus on training emotional coping and stress-reducing strategies but differ in the course content to achieve emotional balance and stress relief (see Materials and methods).

The current study builds on prior work by providing an empirical structure for understanding the mechanisms by which MT reshapes the sense of fairness and cooperation in economic exchanges (Kirk et al., 2011). While cooperative behavior may provide a platform whereby social reputation invites norm-violating behavior (Fehr and Fischbacher, 2004), an understanding of the underlying neural mechanisms by which MT potentially interacts with prosocial behavior may have clinical implications and benefits for patients with psychopathologies such as psychosis, which is characterized by an elementary lack of trust in others (Gromann et al., 2013); social phobia (Sripada et al., 2013); or in patients showing a failure to cooperate, such as borderline personality disorder (BPD) patients (King-Casas et al., 2008).

Previous neuroimaging experiments have identified neural correlates of social exchange games (King-Casas et al., 2005, Rilling et al., 2002, Sanfey et al., 2003). For example, studies using the Ultimatum Game demonstrate that people reject unfair monetary splits even at a cost to themselves, e.g., offers of 20% are rejected about 50% of the time (Camerer, 2003). Yet rational accounts predict that responders would accept any non-zero offer. Presumably, this reflects an emotional reaction to unfairness that overrides rational economic decision processes. The elevated rejection rate of unfair offers is associated with anterior insula activity that scales inversely with offer size and predicts whether an unfair offer is rejected (Sanfey et al., 2003). The anterior insula is a brain area linking interoception (Craig, 2002, Craig, 2009, Critchley et al., 2004) with emotional awareness (Gu et al., 2013). It has recently been proposed that interoceptive function also contributes to value-based decision making (Gu and FitzGerald, 2014, Kirk et al., 2011, Kirk et al., 2014; but see Paulus, 2007). For example, Kirk et al. (2011) show that experienced meditators are less likely to reject unfair offers during the Ultimatum Game and show a shift in activation during unfair offers from anterior to posterior insula, as compared with a non-meditator control group. Similarly, studies show a link between activity in the middle and posterior portions of the insula cortex and mindfulness training (Farb et al., 2007, Kirk et al., 2014) as well as compassion meditation (Lutz et al., 2009).

While no study has directly evaluated the effects of mindfulness training in a longitudinal design on cooperative behavior, recent work has studied the effects of compassion training on prosocial behavior (Klimecki et al., 2013, Leiberg et al., 2011, Weng et al., 2013). However, in these studies, direct effects of altruistic punishment during social exchange (for a review see Rilling et al., 2008) or specific effects of MT per se was not examined.

In the current study, we hypothesize that MT will promote cooperative behavior during social exchanges. A direct advantage of employing a longitudinal design is that it allows us to compute a *cooperation index* reflecting within-subject changes to cooperative and prosocial decision making. Specifically, we predict that MT will increase subjects' cooperation index in the Ultimatum Game. We further predict that MT will modify neural activity in anterior insula and scale inversely with offer size (Sanfey et al., 2003, Kirk et al., 2011). In addition, based on previous research showing that the septal region during social exchange encodes altruistic, prosocial behavior, and unconditional trusting behavior (Harbaugh et al., 2007, Moll et al., 2006, Krueger et al., 2007), we hypothesize that neural signatures in the septal region will be engaged as a function of MT in contrast to CT and provide neural support to the behavioral hypothesis of increased acceptance rates in the MT group.

## Materials and methods

### Subjects

Fifty-one healthy volunteers participated in the Ultimatum Game. The CT group consisted of 24 subjects (20 Caucasians; 4 Afro-Americans) and the MT group consisted of 27 subjects (22 Caucasians; 5 Afro-Americans). The CT group included 13 women and 11 men (mean age 31.1; st.d 9.8), while the MT group included 14 women and 13 men (mean age 32.2; st.d 10.4). The two groups did not differ in terms of mean age or gender distribution. One subject from the MT group was excluded from the analyses based on outlier behavior in the Ultimatum Game (> 3 SD from the mean).

The study was framed as a stress-management program lasting 8 weeks and recruitment procedures consisted of advertising for participants “who want to learn to deal with stress issues in everyday life.” This recruitment strategy was employed in order to reduce self-selection bias to ensure volunteers from a broad demographic range. Subjects were recruited with the understanding that the study consisted of comparing two equally valid stress reduction interventions. In addition, subjects were notified that they would be assigned to a stress reduction intervention in a random manner, which eliminated any self-selection effects between the two interventions. The study was advertised for staff and students around campus at Virginia Tech, Virginia, USA. This recruitment strategy resulted in 238 volunteers who signed up for the study. Of this initial number, 45 subjects were found to be ineligible (33 subjects were using psychiatric medication or had a medical history of psychiatric medication; 12 subjects were MRI ineligible due to either metal implants, claustrophobia, or subjects who had previously suffered from concussions that included a loss of consciousness for more than 10 min). An additional exclusion criterion for the study was prior experience (i.e. regular practice) with mindfulness meditation. All subjects had normal or corrected-to-normal vision, and none in the included cohort had a history of neurological or psychiatric disorders. The non-selected volunteers were put on a waitlist to participate in future studies involving stress-management training. The subjects who were included in the current study were subsequently randomly assigned to receive either MT or CT. Subjects received compensation for their participation according to the following payment scheme: subjects were paid $20 for attendance in each of the 8 weekly group sessions independent of group modality (MT/CT). Subjects were paid according to the decisions they made on three randomly selected rounds during the Ultimatum Game to make sure that subjects made motivated choices during the task, which is a standard procedure in the field of neuroeconomics (Montague and Berns, 2002). Subjects were informed about this payment method prior to the experiment. The subjects received compensation associated with the Ultimatum Game fMRI-task immediately after each scanning session. However, attendance compensation for the 8 weekly group sessions was paid in total upon study completion. All procedures were conducted in accordance with the Institutional Review Board of Virginia Tech.

### Experimental procedures

**Fig. 1:**
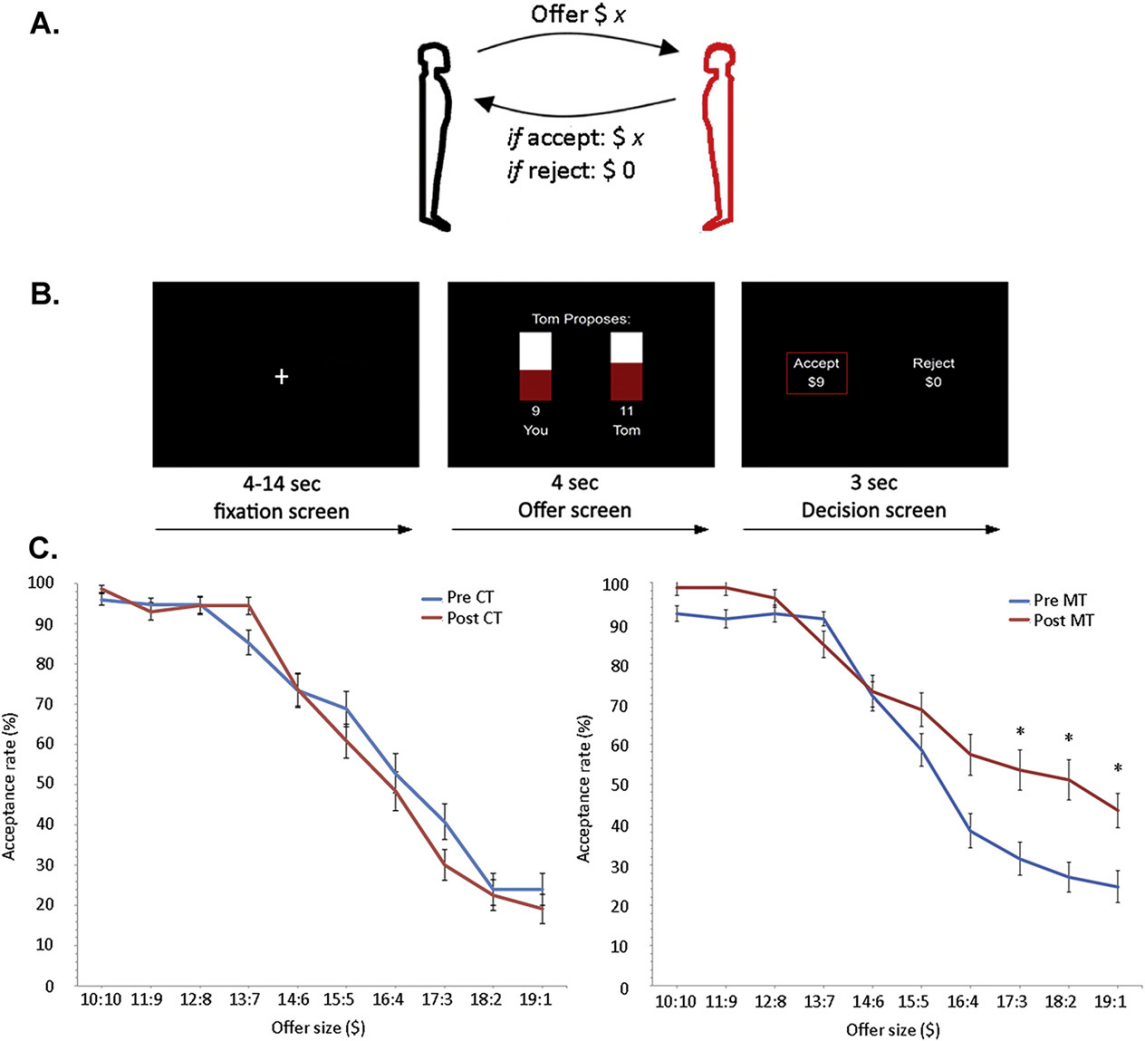
A) 50 subjects played responders in the Ultimatum Game. Subjects choose on each round to accept or reject a monetary split of $20 made by a new partner on each round. 24 subjects in the CT group and 26 subjects in the MT group were scanned pre and post an 8-week mindfulness (MT) or active control training (CT) intervention. B) Trial outline for a single round of the Ultimatum Game. Each trial started with a jittered fixation period (4–14 s) followed by an offer to split $20 (4 s). Finally, subjects indicated the decision to accept or reject the offer (3 s) by pressing one of two buttons on a button box. A red box highlighted the choice being made on each trial. C) Behavioral results from the Ultimatum Game. MT displayed significantly elevated acceptance rates for the most unfair offers ($19:1–$17:3). By contrast, CT did not result in changes in acceptance rates pre and post the 8-week training intervention. The mean and SEM are plotted.

Participants played responders during 40 rounds of an anonymous version of the Ultimatum Game. Prior to scanning, participants were instructed in the task and were subsequently given a test to ensure that the nature and rules of the game were understood participants. The offers were splits of $20. On each round, the participants saw a bar graph with an offer (e.g. “Tom proposes: $9 you $11 Tom”) (Fig. 1). Different names were used on every trial both in the pre-training scanning session as well as in the post-training scanning session. The offer screen was of 4 s duration. Next, the participants were presented with the choice: “Accept ($9) Reject ($0),” which was presented for 3 s in which subjects made a response using a button-box. A red box placed around one of the choices indicated that a decision was made. Finally, a jittered inter-trial interval was presented (4–14 s). Participants had a button-box in each hand and were instructed to press with either left or right hand corresponding to the preferred choice, which was presented on left and right side of the screen. The position of the “accept” and “reject” choices on either left or right side was held constant within subjects and counterbalanced across subjects. Participants were presented with the 40 offers/trials in a predetermined fashion: 5 × $19:1, 5 × $18:2, 5 × $17:3, 5 × $16:4, 5 × $15:5, 3 × $14:6, 3 × $13:7, 3 × $12:8, 3 × $11:9, 3 × $10:10. The sequence of offer presentations was randomized across participants.

The stimuli were presented at a screen resolution of 1024 × 768 pixels. Stimuli were presented and responses collected using NEMO (Human Neuroimaging Lab, Baylor College of Medicine). The stimuli were back-projected via an LCD projector onto a transparent screen positioned over the subjects' head and viewed through a tilted mirror fixed to the head coil.

Prior to the experiment, participants were told that the offers presented had been made by proposers in a previous experiment, i.e. that the offers were real, and that proposers would be paid according to the decision made by the participants. This served as a cover story to enhance the ecology of the game, i.e. that the choices made by subjects had actual consequences for partners on each round of the game. In reality, all the proposals were predetermined similar to other neuroimaging studies using the Ultimatum Game (Sanfey et al., 2003, Crockett et al., 2008, Koenigs and Tranel, 2007, Knoch et al., 2006, Kirk et al., 2011).

Subjects were scanned both before the 8-week training intervention and immediately (i.e. 1–5 days) after the intervention was completed.

### Psychometric data

All volunteers completed the International Positive and Negative Affect Schedule Short Form (I-PANAS-SF) and the Five Facet Mindfulness Questionnaire (FFMQ) at baseline and post-intervention. The I-PANAS-SF is a 10-item version of the mood-assessing PANAS inventory, with similar psychometric properties (Thompson, 2007). The FFMQ is a self-report instrument targeting five aspects of mindfulness: observing, describing, acting with awareness, non-judging of inner experience, and non-reactivity to inner experience (Baer et al., 2008). We report only the total FFMQ score due to data loss of the original hard-copy data, and the fact that only the electronically stored total scores were available for the analysis.

### Procedure for MT

The MT consisted of 8 weeks of practice of mindfulness using a modified version of a mindfulness program titled Mindfulness Based Stress Reduction (MBSR) (Kabat-Zinn, 1990). The modification consisted of reducing the required home practice, which is 45–60 min daily in the canonical MBSR program (Kabat-Zinn, 1990), to 20 min daily. This modification was made to match the daily homework in the active control group and to enhance adherence to the program. A certified MBSR instructor taught the MT program. A structured group format (*n* = 26) was applied whereby participants attended weekly group sessions that introduced them to formal meditation practices and to moment-to-moment, and non-judgmental awareness. Each group session lasted 2.5 h. The MT program also included a full day of meditation between the sixth and seventh meeting sessions. Participants were required to attend at least seven of the eight group sessions and the full-day session to be considered compliant with the training protocol. In addition to group meetings, participants were asked to practice meditation on non-class days for 20 min a day with the assistance of guided meditation CDs. The formal meditation practices included breath monitoring, body scans, and attention to sounds, thoughts, feelings, and bodily sensations. Participants were instructed to maintain a daily log of practice completion, which was collected by the course instructors at every weekly session. In addition to class attendance, participants were required to complete at least 50% of the recommended daily homework. All subjects in the MT group completed the 8-week training program including meeting the minimum training requirements (class attendance and daily homework).

The average daily amount of time spent on home exercises as measured by daily practice logs was 13.6 min (SD = 5.7) for the MT group.

### Procedure for CT

A structured group format was employed for the active control training (CT) intervention, whereby participants attended weekly group sessions with a certified instructor introducing them to progressive muscle relaxation. The weekly sessions were 2.5 h in duration and included 30 min of stretching and exercise. These exercises could be easily completed in comfortable clothing. Some positions could be performed while seated. The exercises were followed by a group discussion for 30 min, with participants sharing their experience on a particular topic and giving updates from previous weeks. Sometimes, the group was asked a question to facilitate conversation, and group members would take turns to answer it. After the group discussion, the facilitator would introduce a new topic; topics included time management, physical activity, sleep, healthy eating, organization, communication, and future goal setting. The facilitator provided information gathered from online sources about each topic. On non-class days, participants were expected to complete their stretching and exercises daily (20 min/day) with the aid of guided practice CDs, and to reflect on the topic for the week. The CT program also included a full day of physical relaxation exercises between the sixth and seventh meeting sessions. Participants were required to attend at least seven of the eight group sessions and the full-day session to be considered compliant with the training protocol. They were instructed to maintain a daily practice log of practice completion, which was collected by the course instructors at every weekly session. In addition to class attendance, participants were required to complete at least 50% of the recommended daily homework.

All subjects in the CT group completed the 8-week training program including meeting the minimum training requirements (class attendance, daily homework).

The average daily amount of time spent on home exercises as measured by daily practice logs was 15.3 min (SD = 6.4) for the CT group.

### fMRI data acquisition

The anatomical and functional imaging was performed using 3 Tesla Siemens Trio scanners. High-resolution T1-weighted scans were acquired using an MPRAGE sequence (Siemens). Functional imaging used an EPI sequence with a repetition time (TR) of 2000 ms, echo time (TE) = 25 ms, flip angle = 90°, 220 mm field of view (FOV), 64 × 64 matrix. Functional slices were oriented 30° superior-caudal to the plane through the anterior and posterior commissures in order to reduce signal drop-out due to magnetic field in-homogeneities (Deichmann et al., 2003). Each functional image was acquired in an interleaved way, comprising 37 4 mm axial slices for measurement of the blood oxygenation level-dependent (BOLD) effect (Ogawa et al., 1990), yielding 3.4 × 3.4 × 4.0 mm voxels.

### fMRI data analysis

Image pre-processing and data analysis were performed using SPM8 (Wellcome Department of Imaging Neuroscience, London, UK). The EPI images were realigned spatially (Friston et al., 1995), corrected for slice timing artifacts, normalized to the Montreal Neurological Institute (MNI) template provided in SPM8, smoothed spatially with an 8 mm isotropic Gaussian kernel, and high-pass filtered in the temporal domain (cutoff period, 128 s). Following pre-processing, a general linear model (GLM) was applied to the fMRI time-series where each stimulus onset, which is offer onset including the 4 s epochs following the offer, was modeled as boxcars and convolved with SPM8’s canonical hemodynamic response function (HRF) (Friston et al., 1996).

A parametric design was used (Buchel et al., 1998) that allowed us to model linear hemodynamic responses using the subject-specific 40 monetary offer trials. First-level GLM analysis included four regressors of interest: 1) pre-MT, 2) pre-CT, 3) post-MT, 4) post-CT for each of the polynomial expansions, using each subject's offer trials in order to model linear parametric modulations of unfairness. Residual effects of head motion were corrected by including the six estimated motion parameters for each subject as regressors of no interest. The mean images from the first level analysis were entered into a second-level, random effects (RFX) analysis accounting for the between subject variance. An ANOVA model using the beta-estimates of the pre- and post-training regressors for each subject according to group modality (MT and CT) for each of the polynomial expansions was applied. Equal variance was not assumed, thus SPM8’s options for non-sphericity correction was applied (Glaser and Friston, 2004). Using t-contrasts allowed us to test for correlations of the fMRI BOLD signal and the parameters of interest performed as linear parametric modulations. The resulting *t* maps were subsequently transformed to *z*-distributions to create a statistical parametric map for each contrast. The statistical results given were based on a single-voxel *t*-statistic or cluster-level corrected corresponding to *p* < 0.05 corrected for multiple comparisons using the false discovery rate statistic (FDR) with an extent threshold of > 10 voxels (unless otherwise stated). The co-ordinates of all activations are reported in MNI space. The data images are displayed using the xjView toolbox.

**Fig. 2:**
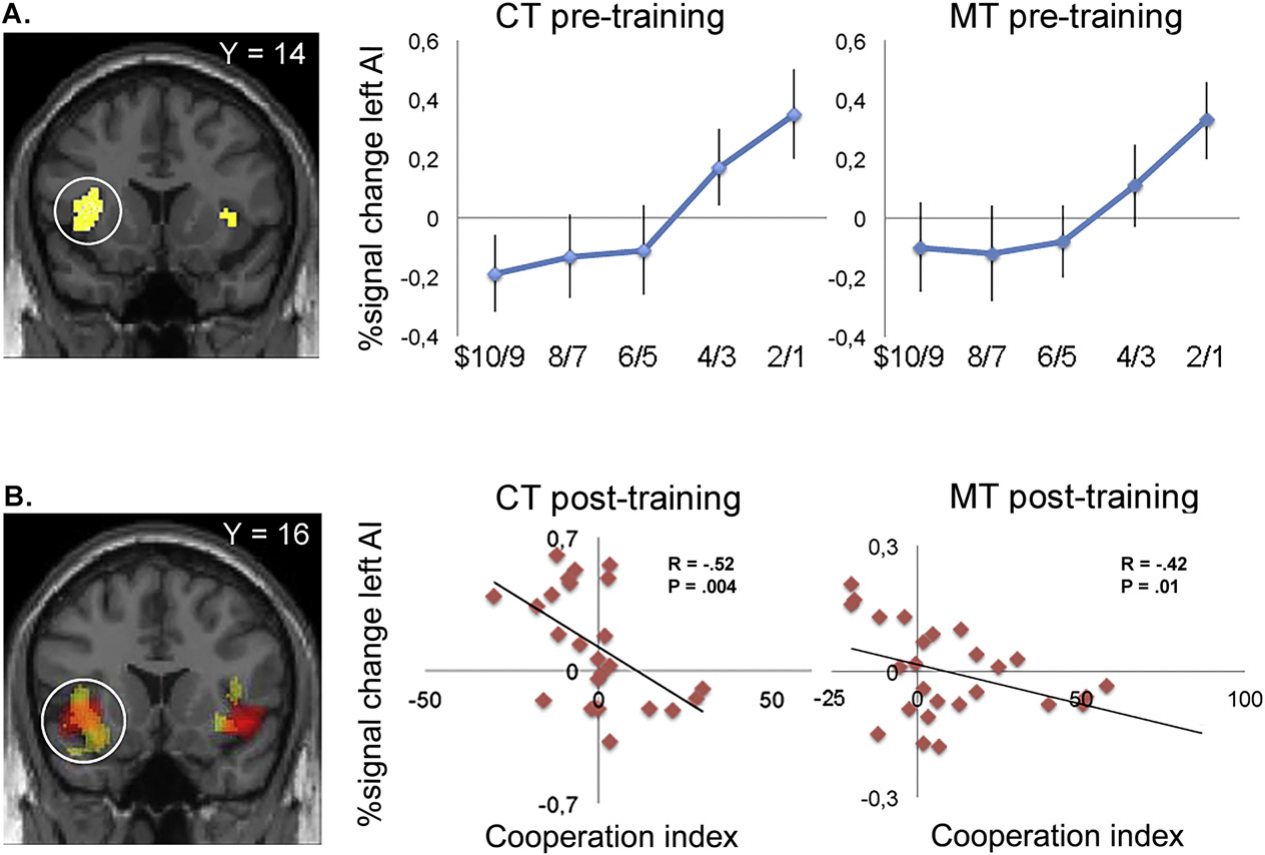
A) Conjunction analysis across groups (MT and CT) in the pre-training condition. Significant conjunction effect between the mindfulness and control group in bilateral anterior insula (AI) displayed at *p* < 0.001, uncorrected. The beta plots from the left AI (circled) display a linear scaling with the size of unfair offers in both the MT group and in the CT group. Mean ± SEM are plotted in increments of $2 bins. B) Cooperation index across groups in the post-training condition. fMRI correlational analysis in the left AI. Regions showing a significant correlation between the cooperation index (calculated as a between-subject measure of the difference in behavioral acceptance rate from pre- to post-training). The left AI is circled and activation maps for both groups are overlaid on a coronal T1-weighted slice displayed at (*p* < 0.005, uncorrected) to illustrate overlap in activation between the two groups (MT group in red; CT group in yellow). Plots of correlation are displayed separately for MT group and CT group. Each data point represents a subject.

In a subsequent analysis, we performed a conjunction analysis to formally establish whether the voxels in the anterior insula scale in both groups in response to unfair offers in the pre-training condition. For the conjunction analysis, we used the pre-MT and pre-CT regressor. The statistical threshold in the conjunction analysis was set at voxel level *p* < 0.05, FDR-corrected with an extent threshold of > 10 voxels. The results are displayed in Fig. 2A.

**Fig. 3:**
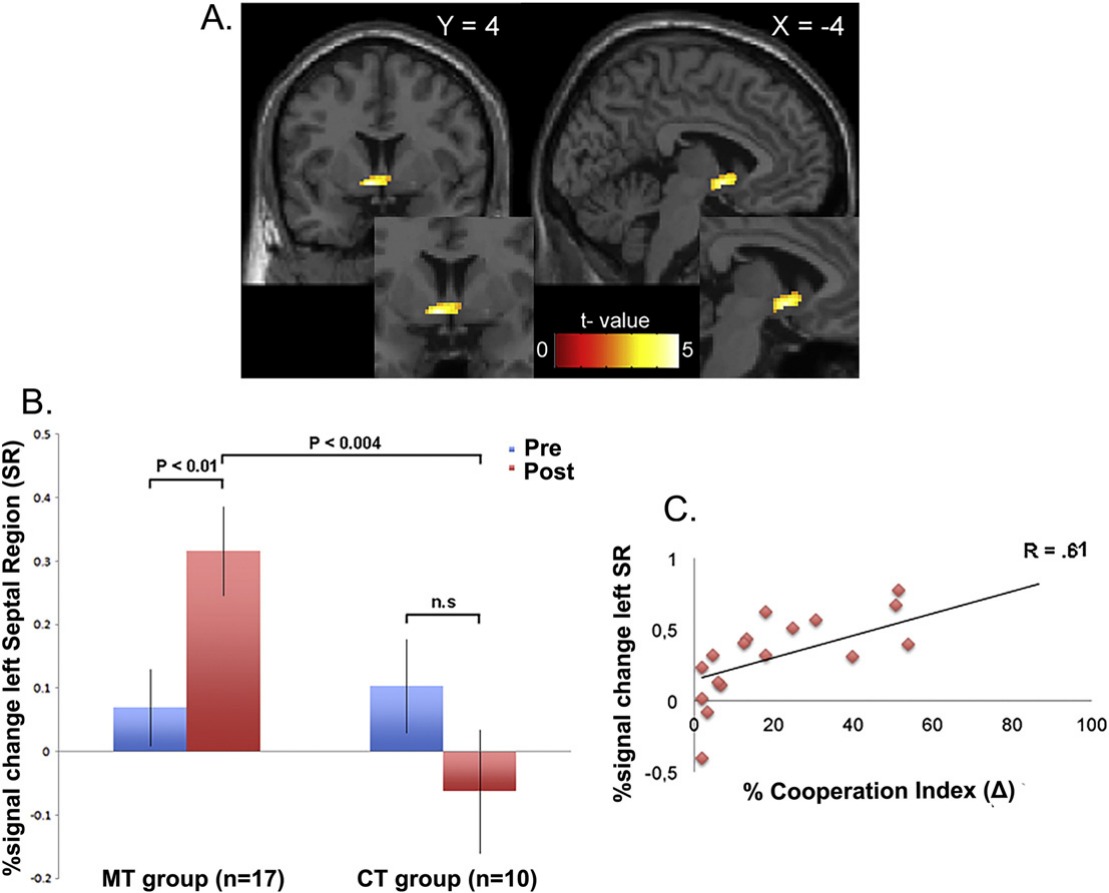
A) Bilateral septal region displayed in coronal and sagittal sections (Left: − 4 4–6; *z* = 4.46; *p* < 0.05, FDR-corrected. Right: 8 16–2; *z* = 3.43; *p* < 0.001, uncorrected). The septal region is more active in the interaction contrast [(Post-MT > Pre-MT) > (Post-CT > Pre-CT)]. No other brain regions reached significance at the whole brain level (*p* < 0.001, uncorrected). B) Region of interest (ROI) in left septal region. Beta values for each group in the ROI (4 mm mask; MNI: − 4 4–6) display higher beta values in the post-MT condition than in both the pre-MT condition as well as in the post-CT condition. Error bars indicate SEM. C) Linear regression showing a positive correlation between a behavioral measure of the individual cooperation index in the 17 subjects in the MT subgroup and activity in left septal region in the post-training condition. Each data point represents a participant.

In a second GLM, we bifurcated the data from subjects exhibiting a positive/increasing and negative/decreasing (or an increasing or decreasing) cooperation index from the two groups (see Fig. S2). Specifically, subjects were subdivided into two categories according to their change in acceptance rates pre- to post-training: 1) subjects who displayed decreased acceptance rates pre- to post-training; 2) subjects who displayed increased acceptance rates pre- to post-training. This procedure yielded 4 regressors for each group MT and CT, respectively: 1) pre-accept increase, 2) pre-accept decrease, 3) post-accept increase, 4) post-accept decrease. Otherwise the analysis was performed using identical parameters as applied in the first GLM described above. The results of this analysis are depicted in Fig. 3, Fig. S4, and Table S2.

A ROI analysis in bilateral anterior insula was identified using the coordinates provided by the pre-training data (Fig. 2A). A spherical mask with a 5 mm radius centered at [− 32 14 14] and [34 10 8] was used to extract the time series from bilateral anterior insula from the post-training data in both groups. A correlation analysis was computed using each subject's cooperation index as a between-subject statistical regressor against the first-order linear parametric regressor in each group (MT and CT). The results emerging from this ROI analysis are displayed in Fig. 2B.

**Fig. 4:**
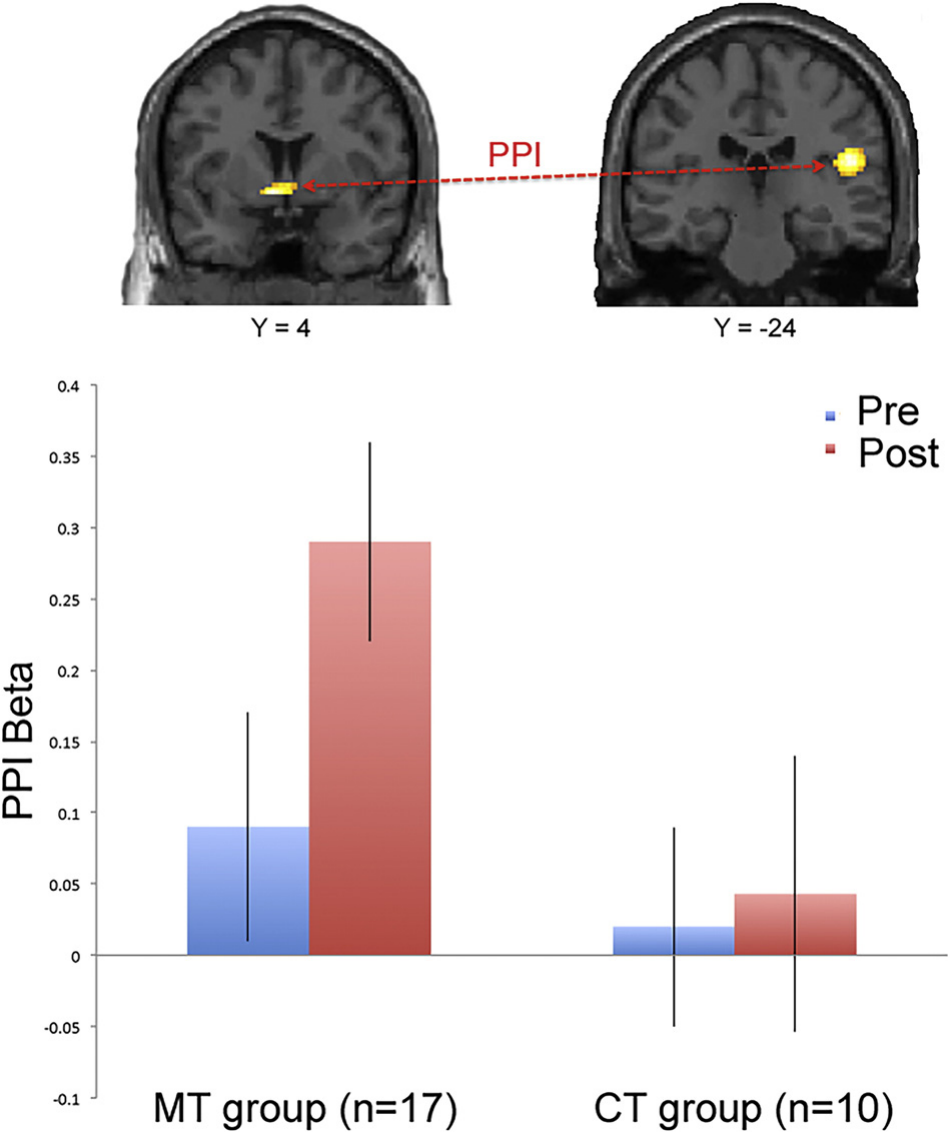
Top: PPI displaying increased coupling between the left septal seed region and the right posterior insula in the MT subgroup (50–26 18; *z* = 3.36; *p* < 0.001, uncorrected). No other brain regions showed suprathreshold activity (*p* < 0.001, uncorrected) in a whole brain analysis. Bottom: Average β-estimates from the right posterior insula measuring the correlation between BOLD activity in the septal region and insula in both MT and CT subgroups in both pre- and post-training conditions. Septal region activity in the CT group did not exhibit significant connectivity with the posterior insula. Error bars are SEM.

For the effective connectivity analysis implemented as psychophysiological interaction analysis (PPI) (Friston et al., 1997), we assessed changes in effective connectivity between the seed region in the left septal region and other brain regions. The PPI employed a regressor representing the deconvolved time series of neural activity within a 5-mm sphere centered on left septal region (MNI coordinates: − 4 4–6), which constituted the physiological variable, a second regressor representing the psychological variable, specifically the first-order linear parametric regressor that scales with unfair offer sizes, and a third regressor representing the cross-product of the previous two (the PPI term). The model also included motion parameters as regressors of no interest. The PPI was carried out in each subject and entered into random-effects analysis separately for each of the two groups. These two groups were the 17 subjects in the MT group and the 10 subjects in the CT group that displayed an increasing cooperation index (Fig. S2) and both time points (pre and post) were included in the analysis. The results from this analysis are displayed in Fig. 4.

### Behavioral results

To assess group differences in behavioral decision making when playing the Ultimatum Game, we employed a repeated-measurement ANOVA (rANOVA) using the Matlab function “fitrm.” Subjects were grouped by their membership in the CT or MT group (1 Factor). Each subject was assigned their pre- and post-acceptance rate measurement for each given offer size and rANOVA was performed for each offer level. We found a significant (*p* < 0.05) increase for the acceptance rate within the MT group (significant positive effect of “MT” grouping factor on acceptance rates in rANOVA for the low end of the offer range ($17:3 −$19:1) and no significant differences at *p* < 0.05 for the other offers (Fig. 1C). In absolute terms, MT was responsible for a dramatic increase in acceptance rates, such that the MT group accepted a $19:1 split on 24% of trials pre-training, compared to 44% post-training. By contrast, the control group accepted a $19:1 split on 24% of trials pre-training and 19% of trials post-training.

We further assessed the training effect using the Five Facet Mindfulness Questionnaire (FFMQ) total score,^1^ again using rANOVA with the CT and MT membership as factor and the pre- and post-FFMQ score of each subject as measurements. We observed a significant (*p* < 0.05) increase in post-training FFMQ scores for both training types (significant intercept effect in rANOVA),;however, we also found an additional significant (*p* < 0.05) increase of post-MT-FFMQ scores over the post-CT-FFMQ scores (significant positive slope of factor “MT” over “CT” in rANOVA). Hence, MT training yielded significantly larger FFMQ increases over CT training.

The I-PANAS-SF did not result in changes. Specifically, we performed rANOVA again, with the same grouping factor “MT” and “CT” and the I-PANAS-SF pre- and post-scores. However, no significant changes were found in this dimension of psychological assessment.

**Fig. S1:**
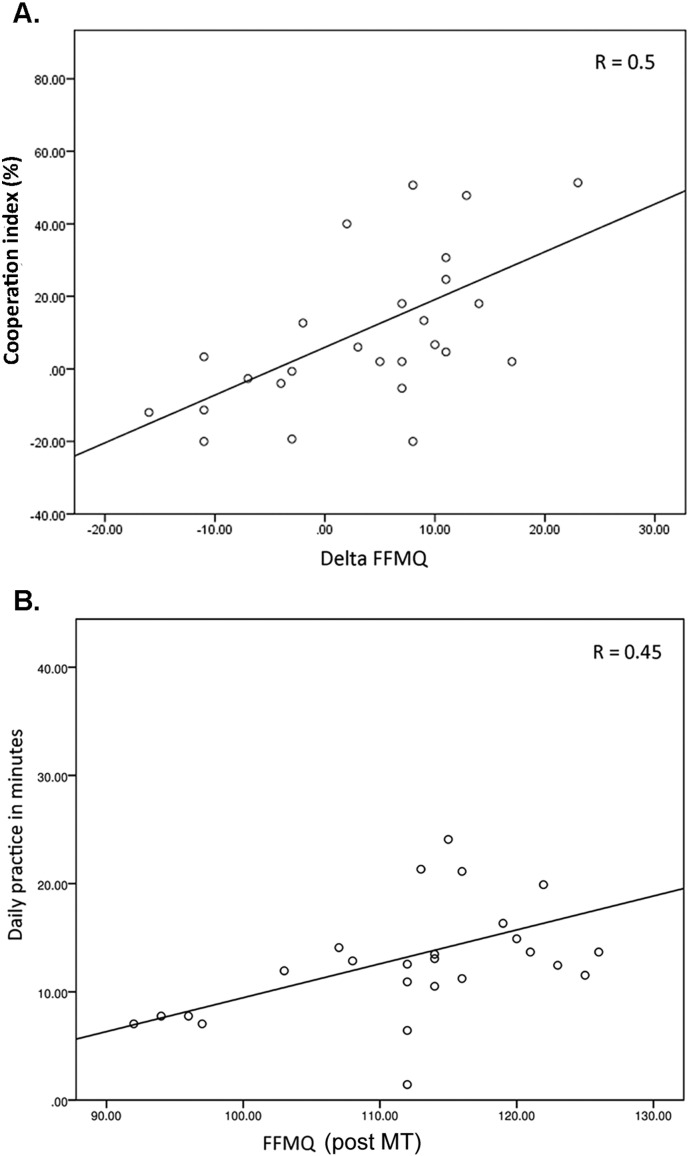
A) Positive correlation in the MT group (*n* = 26) between the cooperation index, which is computed as the within-subjects measure of the difference in percent acceptance rate of monetary offers from pre-intervention to post-intervention, and the delta value of each individuals' FFMQ score [post-MT > pre-MT]. This relationship was not evident in the CT group (*R* = − 0.21; *p* < 0.1). B) Positive correlation between the FFMQ and daily home practice in the MT group. The average daily amounts of time spend on home exercises as measured by daily practice logs was 13.6 min (st.d. = 5.7) for the MT group. The CT group spent an average of 15.3 min (st.d. = 6.4) on home exercises as measured by daily practice logs, albeit the CT group did not display a significant correlation between FFMQ and home practice (*R* = 0.11; *p* < 0.3). Note that daily practice logs from two subjects in the MT group were not collected due to technical issues and hence could not be included in this behavioral analysis (they were however both included in the neural analysis).

In order to quantify if MT was related to the decision to accept or reject monetary offers, we computed the difference of each individuals' FFMQ score [post-MT > pre-MT] as well as the difference in acceptance rates [post-MT > pre-MT]. We phrased this difference in acceptance rate as the *cooperation index*, which represents the within-subject changes in acceptance rates post-training. The data showed a positive correlation between an increase in the FFMQ score and an increase in acceptance rates after MT (Pearson's *R* = 0.50; *p* < 0.004) (Fig. S1 A). In the CT group, we found no significant relationship between subjects' cooperation index and subjects' differences in FFMQ delta scores (*R* = − 0.21; *p* < 0.1).

Finally, FFMQ scores post-MT displayed a positive correlation with amount of time spent on daily mindfulness home practice (*R* = 0.45; *p* < 0.01) (Fig. S1B). There was no significant relationship between the daily home practice and the FFMQ in the CT group (*R* = 0.11; *p* < 0.3). There was also a significant difference across treatment conditions in accumulated total monetary earnings during the Ultimatum Game (Fig. S6). The MT group increased their earnings significantly compared to pre-training (paired *t* = 2.07; *p* < 0.04). The CT group did not display significant differences across the intervention. We did, however, not observe a significant correlation between practice time and acceptance rates in the MT group (*R* = 0.2; *p* < 0.1).

### Neuroimaging results

#### 2.9.1 Pre-training assessment of anterior insula activity to unfair offers

Based on previous studies (Kirk et al., 2011, Sanfey et al., 2003), we hypothesized that the anterior insula should encode the responders' negative emotional response to unfair offers in both groups (MT and CT) in the pre-training condition. We performed a conjunction analysis using the MT (*n* = 26) and the CT group (*n* = 24) separately from the pre-training condition. The conjunction analysis was implemented using the subject-specific first-order linear parametric regressor that scales with unfair offer sizes. In a whole brain analysis, we found activity in bilateral anterior insula (Left: − 32 14 14; *z* = 4.86; *p* < 0.05, FDR-corrected. Right: 34 10 8; *z* = 3.50; *p* < 0.001, uncorrected) (Fig. 2A) among other regions (Table S1).

#### Post-training changes anterior insula activity to unfair offers

In the post-training condition, we hypothesized that anterior insula should encode a response to unfair offers that would scale with the subject-specific *cooperation index* (see Behavioral Results). Accordingly, subjects displaying a decreased cooperation index should exhibit elevated anterior insula activity post-training, presumably reflecting the responders' negative emotional response associated with unfair offers (Sanfey et al., 2003). To formally test this conjecture, we computed each subject's cooperation index as a between-subject statistical regressor separately for MT (*n* = 26) and CT (*n* = 24) against the first-order linear parametric regressor as an ROI analysis in bilateral anterior insula (see Materials and methods). We observed a significant correlation between a decreasing cooperation index and increased activity in bilateral anterior insula in both the MT group (Left: *R* = − 0.42; *p* = 0.01) and the CT group (Left: *R* = − 0.52; *p* = 0.004) (Fig. 2B). The difference of the regression slopes between the two groups can be explained by the variability in acceptance rates between the two groups. After conversion of the Pearson's R into z scores, we found that the slopes between the two groups were significantly different (*p* < 0.05). The elevated activation profile of the anterior insula post-training in those subjects who displayed a decreasing cooperation index may suggest that the negative emotions that arise when social fairness norms are breached, elicit a robust endurance or repetition effect. As such, this result has important implications in that it suggests that the MT group has greater ability to effectively regulate anterior insula and thereby promote cooperative decision making.

#### Neural assessment of cooperative behavior as a function of training condition

**Fig. S2:**
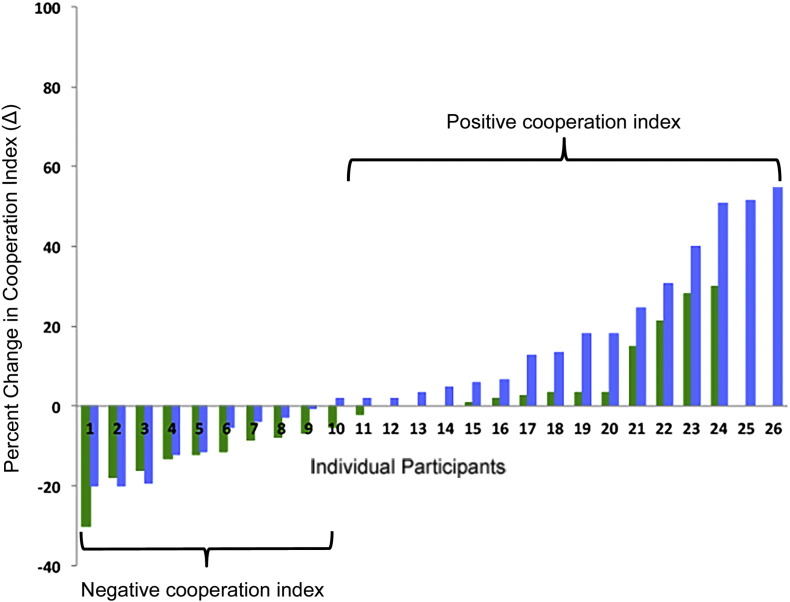
Cooperation index across groups. Blue indicates MT group (*n* = 26). Green indicates CT group (*n* = 24). 17 subjects in the MT group displayed a positive cooperation index, whereas this number for the CT group was 10. There were 9 subjects in the MT group who displayed a negative cooperation index and 11 in the in CT group. 3 subjects in the CT group did not display a change in delta.

**Fig. S3:**
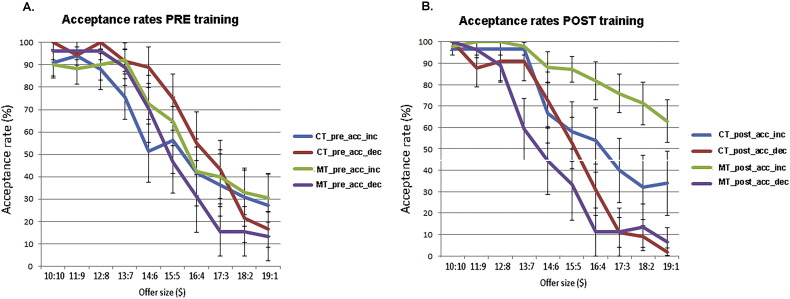
Subgroup division of behavioral responses during Ultimatum Game. A) Acceptance rates in the pre training version for both groups (CT and MT). There are no significant differences between the subgroups in the pre training. The subgroups are: CT and MT divided into subjects who displayed an increased cooperation score [MT_acc_inc (*n* = 17); CT_acc_inc (*n* = 10)] and those who exhibited a decreased cooperation score [MT_acc_dec (*n* = 9); CT_acc_dec (*n* = 11)]. B) Acceptance rates in the post-training version for both groups. As there were no differences in the pre training version between any of the subgroups, we can infer that the emergent behavioral differences in acceptance rates in the post training condition is a function of the intervention. Error bars are SEM. acc_dec = acceptance rate decrease; acc_inc = acceptance rate increase.

We next asked the question which brain regions are associated with the increased tendency to accept an unfair offer post-MT relative to post-CT. In order to account for the variability in acceptance rates between the two groups, we subdivided subjects into those who exhibited an increasing cooperation index post-training vs. those who displayed a decreasing cooperation index. This subdivision yielded 17 subjects, or 65% from the MT group, who displayed an elevated cooperation index post-MT, whereas 10 subjects, or 41% exhibited an increasing cooperation index in the CT group (Fig. S2). Furthermore, Fig. S3 show that the subdivision does not account for pre-existing differences in percent acceptances rates prior to the 8-week interventions, but rather are emergent behavioral differences as a function of the training intervention. Similarly, we did not observe pre-existing differences in the neural data across the MT and CT groups (Fig. S5). It is noteworthy that the cooperative MT group and the cooperative CT group did not differ significantly in either age or gender.

The key contrast based on this subdivision is the interaction between training condition and time: [(Post-MT > Pre-MT) > (Post-CT > Pre-CT)]. Importantly, this interaction was balanced with respect to decision type (accept or reject) between groups (MT and CT). Consequently, the interaction contrast allowed us to identify brain regions that were uniquely mediating the effect of suspending fairness goals in the MT group compared to the CT group.

Note that reasons for subdividing (or dichotomize) the data are to obtain a balanced interaction analysis, that is, dividing the subset of cooperative subjects from MT and CT groups who do not differ in terms of behavioral responses to unfairness. Hence differences between groups in the neural output cannot be attributed to behavioral differences, but only to differences in the underlying strategies in choosing a more cooperative behavioral output.

This analysis revealed significant activity in a cluster of voxels with peak coordinates in the left septal region, and extending to include the adjacent head of the caudate nucleus bilaterally (Left: − 4 4–6; *z* = 4.46; *p* < 0.05, FDR corrected. Right: 8 16–2; *z* = 3.43; *p* < 0.001, uncorrected) (Fig. 3A).

To ensure that the activation in the septal region was driven by a significant increase in the MT group post-training, we inspected the average β-values from the left septal region (MNI: − 4 4–6). We found that the MT group displayed increased activation from pre- to post-training (paired *t* = 2.59; *p* = 0.01). Importantly, this pattern was not present in the CT group (paired *t* = − 1.3; *p* = 0.19). The post-training condition showed a significant difference between the MT and CT group (paired *t* = 3.14; *p* = 0.004) (Fig. 3B).

 Furthermore, in a linear regression analysis, we estimated the impact of the left septal region against a behavioral measure of each individual**'**s cooperation index in the cooperative MT subgroup. Subjects who showed the highest cooperation index also showed the highest activation in the left septal region in the post-training condition (*R* = 0.61; *p* = 0.001) (Fig. 3C).

**Fig. S4:**
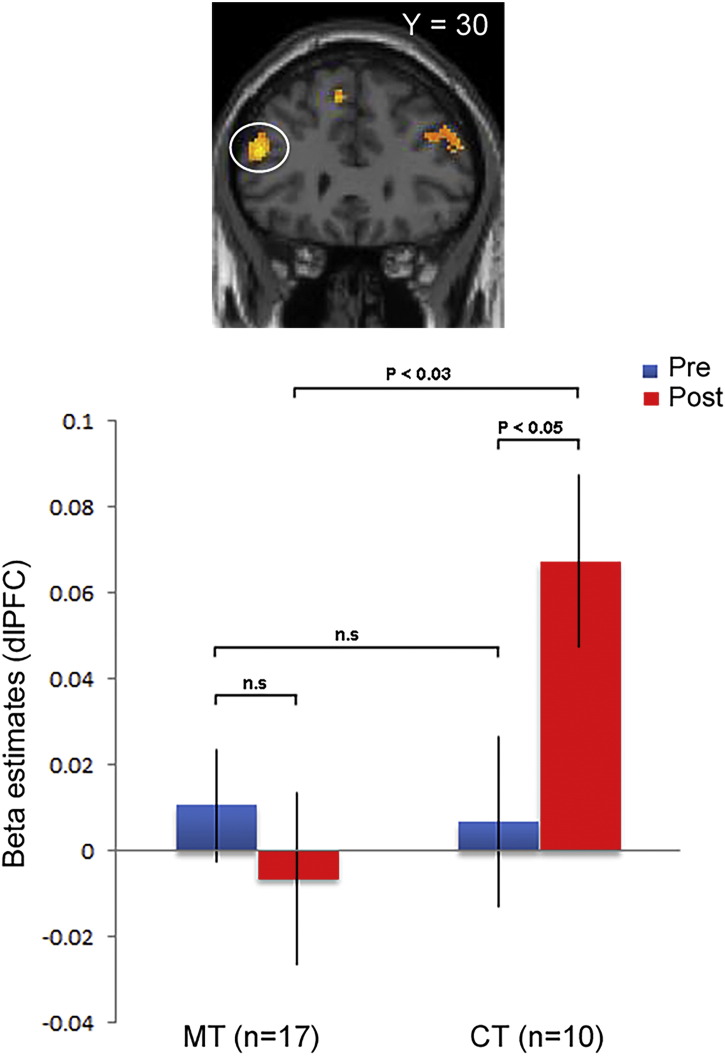
Subdivision RFX analysis of subjects in the increasing cooperation group. Top: Bilateral DLPFC displayed in coronal sections at *p* < 0.005, uncorrected (See Table S2). Bilateral DLPFC is more active in the interaction contrast [(Post CT > Pre CT) > (Post MT > Pre MT)]. Bottom: Region of interest in left DLPFC. Average beta values extracted for each group in the defined ROI (4 mm mask; MNI: − 48 32 22) display higher beta values in the post-CT condition than in both the pre-CT condition as well as in the post MT condition. Error bars indicate SEM.

We expected that cooperative controls would recruit the dorsolateral prefrontal cortex (DLPFC) as previous studies have demonstrated that the DLPFC reduces subjects' willingness to reject unfair offers in the Ultimatum Game (Knoch et al., 2006, Sanfey et al., 2003). Indeed, in the reverse interaction [(Post-CT > Pre-CT) > (Post-MT > Pre-MT)], we observed activity in bilateral posterior parietal cortex and bilateral dorsolateral prefrontal cortex (Fig. S4) among other regions (Table S2 for a complete list of activations), albeit only when lowering the significance threshold considerably (*p* < 0.005, uncorrected). Due to the low threshold, we report these results for completion only as supplementary material (Table S2).

#### Functional connectivity between septal region and posterior insula in the MT group

As we found support for the hypothesis that the septal region plays a mediating role in the decision process to accept or reject an unfair offer in the cooperative MT group in the GLM analysis, we subsequently employed a connectivity analysis to assess the physiological coupling between the septal region and other brain regions in a whole brain analysis. Based on our previous study (Kirk et al., 2011) where we identified increased posterior insula activity in the presence of an unfair offer in expert meditators, we speculated that the MT group might exhibit an increased connectivity between these two brain regions in the context of unfair offers. To test this hypothesis, we performed a functional connectivity analysis with psychophysiological interactions (PPI) (Friston et al., 1997) using the left septal region as the seed region. Specifically, we assessed if the physiological coupling between the septal regions and the posterior insula changed relative to a modulation in the psychological parameter. The MT group showed a strong increase in connectivity between the septal region and the right posterior insula (50–26 18; *z* = 3.36; *p* < 0.001, uncorrected). No other regions emerged in a whole brain analysis at *p* < 0.001, uncorrected. There was no significant voxels (*p* < 0.001, uncorrected) in the CT group (Fig. 4). This result suggest that the increased connectivity between the septal region and the posterior insula is unique for the cooperative MT group and appears essential for these subjects' decision to accept an unfair offer.

## Discussion

The results from this study extend our knowledge of the neural basis of cooperation in a socioeconomic context by suggesting that prosocial behavior may be promoted through psychological behavioral interventions, such as MT. The results expand previous work (Kirk et al., 2011) by employing a randomized controlled design using MT and active CT to study the impact an 8-week behavioral intervention (MT or CT) exerts on decision-making processes in the context of the Ultimatum Game (Guth et al., 1982, Bolton and Zwick, 1995). MT showed a profound effect on subjects' acceptance rates in the Ultimatum Game. Subjects in the MT group were, after 8-weeks of training, willing to accept significantly more unfair offers than subjects in the CT group.

Ultimatum Game behavior resulted in collectively higher earnings for MT subjects (responder role) as well as for their social partners (proposer role). However, when playing a CT subject, there were no increases in collective earnings (Fig. S6). Thus, personal as well as social partners' monetary earnings increased with MT, which suggest a prosocial benefit of MT.

Further validation that MT was responsible for this behavioral profile was substantiated by individual FFMQ scores showing 1) a higher score in the MT group compared to the CT group in the post-training condition, 2) a positive correlation between increasing acceptance rates from pre- to post-training and FFMQ scores in the MT group but not the CT group (Fig. S1 A), and finally, 3) a correlation between weekly home practice and the FFMQ in the MT group but not the CT group (Fig. S1B).

The behavioral findings in this study resonate with recent results that have demonstrated changes in decision making in the Ultimatum Game following both affect induction (Harlé and Sanfey, 2007, Harlé et al., 2012) and emotional reappraisal (van't Wout et al., 2010, Grecucci et al., 2013). Similar results have been obtained with neurophysiological techniques, such as TMS (Knoch et al., 2006). However, our study extends these findings in important ways by demonstrating that MT over the course of 8 weeks leads to prosocial behavioral changes compared to CT.

**Fig. S5:**
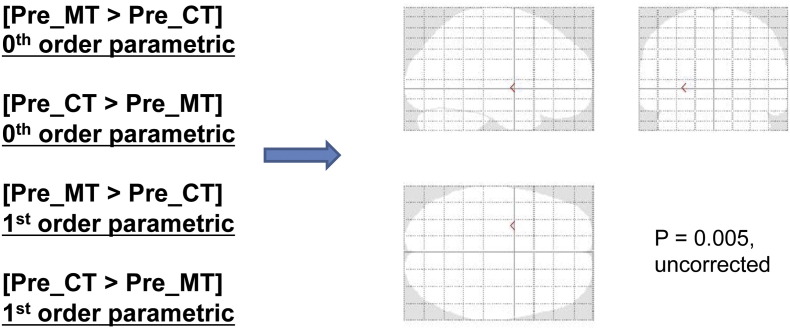
There are no pre-existing differences across the two groups when looking at both the 0th order and 1st parametric regressors (*p* < 0.005, uncorrected). This null result verifies that our analyses do not confound any pre-existing differences between the groups.

On a neural level, we found that anterior insula in the pre-training condition in both groups was elevated in the context of unfair offers in accordance with previous findings (Sanfey et al., 2003). Post-training, we observed that those subjects who displayed a decreasing cooperation index exhibited increased anterior insula activity (Fig. 2B). This result suggests that regulation of the anterior insula in the context of social exchange promotes an increased ability to accept unfair monetary offers. This notion is reflected in anterior insula activity where we found that the regression slope in the MT group was significantly lower compared with the CT group. Previous results support this result where it was found that anterior insula was attenuated when presented with unfair offers in a meditation group (Kirk et al., 2011). These findings fit nicely with theoretical accounts of mindfulness practice, whereby MT does not reduce negative emotions from arising (i.e. note that the MT group exhibit a significant correlation between acceptance rates and anterior insula activity; Fig. 2B) but rather enables an increased ability to effectively regulate emotions by not acting on them (Chiesa et al., 2013). This observed pattern in the anterior insula cannot be ascribed to pre-existing behavioral (Fig. S3) or neural differences (Fig. S5) but seems to arise as a result of the training intervention. As we found a neural bifurcation in the anterior insula during unfair offers in the CT and MT subgroups, it rules out the possibility that these effects merely reflect repetition effects.

Why did some subjects in the MT group fail to show a transfer of the MT regimen to an elevated behavioral acceptance rate of unfair offers? One plausible explanation might be a lack of interest during training. However, data from home practice (two sample *t* = − 0.67, *p* < 0.5) and class attendance (two sample *t* = − 1.2, *p* = 0.2) showed equal training engagement in the subjects in the MT group that displayed an increased cooperation index compared to those who displayed a decreased cooperation index. An alternative explanation could be that subjects in the MT group that showed a negative cooperation index compared to those in the MT group who showed a positive cooperation index started off with lower mindfulness scores as assessed by the FFMQ. However, the data did not show evidence of such a pattern (two sample *t* = 0.25, *p* = 0.7). A final explanation might be that pre-existing neural differences drives the transfer effects, albeit we did not find neural difference in the two subgroups prior to training.

The acceptance of norm violating behavior evident in the MT group is unorthodox in the existing literature on fairness sensitivity (Fehr and Gachter, 2002, Fehr and Fischbacher, 2003) and furthermore contrasts the involvement of the anterior insula in the context of violation of social norms and mistrust in social interaction paradigms (Rilling et al., 2002, Spitzer et al., 2004, King-Casas et al., 2005, King-Casas et al., 2008, Montague and Lohrenz, 2007, Xiang et al., 2013, Sanfey et al., 2003). This leaves open the possibility that MT may be applied as a psychological training tool in psychopathologies such as BPD (King-Casas et al., 2008) and MDD (McGrath et al., 2013) where regulation of the anterior insula in particular may be essential to ameliorate disease symptoms.

In order to address the question which neural regions reduce the urge to reject an unfair offer in the MT and CT group we isolated the subjects in the CT and MT groups that displayed an elevated tendency to accept an unfair offer in the post-training session compared to the pre-training session. We found that the bilateral septal region (Fig. 3) was more active in the MT group in the post-training condition when presented with an unfair offer compared to the CT group. The septal region is a limbic region that has been associated with a range of social behaviors. For example, the septal nuclei contains neuropeptides such as oxytocin and vasopressin (Loup et al., 1991) that are involved in pair bonding (Young and Wang, 2004). Interestingly, studies have shown that administration of oxytocin in humans increased cooperation in an economic social exchange paradigm (Kosfeld et al., 2005). Furthermore, this region is involved in unconditional economic trust in the social domain (Krueger et al., 2007) and altruistic and prosocial behavior (Harbaugh et al., 2007, Moll et al., 2006). In one study, the septal region encoded the automaticity of empathy irrespective of emotional directionality, suggesting that even negative emotions activate the septal region and may drive empathic prosocial motivation and subsequent prosocial behavior (Morelli et al., 2012).

It is noteworthy that the activation of the septal nuclei was found in a linear parametric regression analysis computing elevated responses as the size of unfair offers increased, that is, as the size of monetary gain to one-self decreased. This suggests that the septal nucleus encodes the voluntary decision to effectively donate a larger monetary reward to a social partner and thus suspends fairness goals. Hence the data do not support an alternative interpretation, namely, that the septal regions and adjoining head of the caudate—which has been consistently involved in anticipation of rewards (Schultz et al., 1997, Lohrenz et al., 2007, Kuhnen and Knutson, 2005)—compute the decision to maximize monetary rewards for oneself, and thus reflect actions based on self-interest.

Previous studies have implicated the DLPFC in reducing subjects' willingness to reject unfair offers in the Ultimatum Game (Knoch et al., 2006, Sanfey et al., 2003). In the subset of participants who primarily accepted unfair offers (i.e. those subjects with increased cooperation index), we found increased activity in bilateral DLPFC for CT only, but not for MT (Fig. S4). This activation pattern might reflect the higher cognitive demands in order to overcome the emotional tendency to reject an unfair offer (Kirk et al., 2011, Knoch et al., 2006, Sanfey et al., 2003). By contrast, the MT group did not recruit the DLPFC to overcome the urge to reject an unfair offer. This result is consistent with studies on meditation on pain processing that found that pain activation was found for meditators within the dorsal ACC, thalamus, and insula. In contrast, controls showed stronger activation in several areas including bilateral dlPFC (Grant and Rainville, 2009, Grant et al., 2010; for a review, see Chiesa et al., 2013).

We observed elevated connectivity between the septal region and the posterior insula during the processing of unfair offers, which was unique for the cooperative MT group. The posterior insula seems to be connected to somatic and visceral inputs and outputs, assisting the interpretation and modulation of the autonomic signals ascending from the body. A model proposed by Craig (2009) argues that the anterior insula is involved in social motivational and cognitive conditions, whereas the posterior part is involved in visceral interoceptive representations. This model is in line with recent findings in the domain of mindfulness, whereby the right posterior insula is involved in focused attention to internal experiences (Hölzel et al., 2008), and momentary self-reference (Farb et al., 2007). Previous results (Kirk et al., 2011) showed activation in the posterior insula suggesting that expert meditators particularly during unfair offers were better able than controls to maintain interoceptive awareness presumably by attending to internal bodily states. This is consistent with the present findings and suggests that MT interact with interoceptive processes in the posterior insula and drives the cooperation index change in the MT group.

In summary, the results from this study suggest that MT modulates behavioral decision strategies during presentation of unfair offers in the Ultimatum Game. The results further provide evidence that the underlying neural mechanisms by which prosocial behavior may be acquired is through a striatal network including the septal region, however future work should try to disentangle the exact role of MT in the underlying self-interested rational vs. prosocial motives of Ultimatum Game behavior. In addition, the results point to effects of MT as an enhanced ability to regulate anterior insula activity, which may be responsible for increased collective earnings during social exchanges. Future research should aim to delineate the potential clinical implications of MT in patients with psychopathologies such as psychosis, which is characterized by an elementary lack of trust in others (Gromann et al., 2013), social phobia (Sripada et al., 2013), or patients with perturbed abilities to cooperate such as BPD patients (King-Casas et al., 2008). Research into the clinical efficacy of MT and other therapeutic approaches may carry major public health significance.

The following are the supplementary data related to this article.

**Fig. S6:**
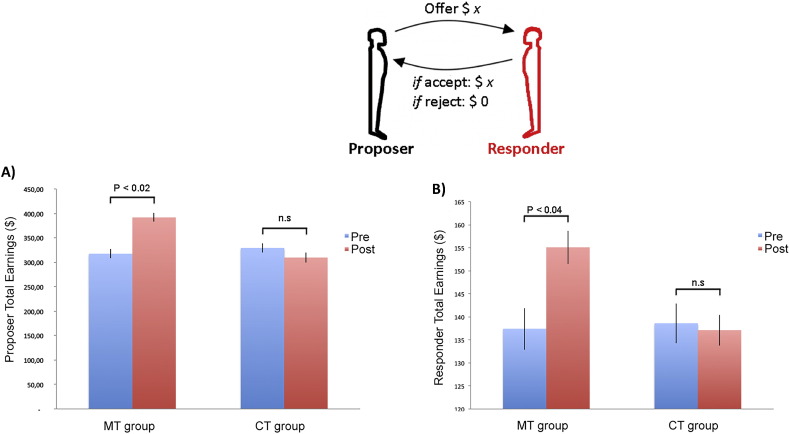
Accumulated total monetary earnings for MT and CT group across the 40 rounds of the Ultimatum Game divided into A) Proposer share and B) Responder share. A) Across all subjects the MT group increased their earnings significantly compared to pre training (paired *t* = 2.07; *p* < 0.04). The CT group did not display significant differences across the intervention. B) The proposer share also increased significantly when a proposer interacted with a subject belonging to the MT group post-training (paired *t* = 2.58; *p* < 0.02) as a opposed to the CT group (paired *t* = 1.6; *p* < 0.1).

Supplementary tables Image 1

## Footnotes

1 Only the FFMQ total scores were analyzed (see Methods section). We note that the results from the FFMQ should be interpreted with caution. Future research may help clarify which aspects of mindfulness (e.g. one or several of the five factors on the FFMQ) might be important in modulating cooperative economic decision making.
