# Supplementary material for: Mindfulness training increases cooperative decision making in economic exchanges: Evidence from fMRI
Source: Neuroimage. 2016 Sep;138:274–83. doi: 10.1016/j.neuroimage.2016.05.075 (PMC4954868; doi:10.1016/j.neuroimage.2016.05.075)
Supplement: Supplementary file 1 — Supplementary tables [file mmc1.pdf]

Supplementary Material

Figure legends

Figure S1

A) Positive correlation in the MT group (n=26) between the cooperation index, which is computed as the within-subjects measure of the difference in percent acceptance rate of monetary offers from pre-intervention to post-intervention, and the delta value of each individuals' FFMQ score [post-MT > pre-MT]. This relationship was not evident in the CT group ($R = -.21$; $p < 0.1$).

B) Positive correlation between the FFMQ and daily home practice in the MT group.

The average daily amounts of time spend on home exercises as measured by daily practice logs was 13.6 minutes (st.d. = 5.7) for the MT group. The CT group spent an average of 15.3 minutes (st.d. = 6.4) on home exercises as measured by daily practice logs, albeit the CT group did not display a significant correlation between FFMQ and home practice ($R = .11$; $p < 0.3$). Note that daily practice logs from two subjects in the MT group were not collected due to technical issues and hence could not be included in this behavioral analysis (they were however both included in the neural analysis).

Figure S2

Cooperation index across groups.

Blue indicates MT group (n=26). Green indicates CT group (n = 24). 17 subjects in the MT group displayed a positive cooperation index, whereas this number for the CT group was 10. There were 9 subjects in the MT group who displayed a negative cooperation index and 11 in the in CT group. 3 subjects in the CT group did not display a change in delta.

Figure S3

Subgroup division of behavioral responses during Ultimatum Game.

A) Acceptance rates in the pre training version for both groups (CT and MT). There are no significant differences between the subgroups in the pre training. The subgroups are: CT and MT divided into subjects who displayed an increased cooperation score [MT_acc_inc (n=17); CT_acc_inc (n=10)] and those who exhibited a decreased cooperation score [MT_acc_dec (n=9); CT_acc_dec (n=11)].

B) Acceptance rates in the post-training version for both groups. As there were no differences in the pre training version between any of the subgroups, we can infer that the emergent behavioral differences in acceptance rates in the post training condition is a function of the intervention. Error bars are SEM.

acc_dec = acceptance rate decrease; acc_inc = acceptance rate increase.

Figure S4

Subdivision RFX analysis of subjects in the increasing cooperation group.

Top: Bilateral DLPFC displayed in coronal sections at $P < 0.005$, uncorrected (See Table S2). Bilateral DLPFC is more active in the interaction contrast [(Post CT > Pre CT) > (Post MT > Pre MT)].

Bottom: Region of interest in left DLPFC. Average beta values extracted for each group in the defined ROI (4 mm mask; MNI: -48 32 22) display higher beta values in the post-CT condition than in both the pre-CT condition as well as in the post MT condition. Error bars indicate SEM.

Figure S5

There are no pre-existing differences across the two groups when looking at both the 0th order and 1st parametric regressors ($P < 0.005$, uncorrected). This null result verifies that our analyses do not confound any pre-existing differences between the groups.

Figure S6

Accumulated total monetary earnings for MT and CT group across the 40 rounds of the Ultimatum Game divided into A) Proposer share and B) Responder share.

A) Across all subjects the MT group increased their earnings significantly compared to pre training (paired $t = 2.07$; $p < 0.04$). The CT group did not display significant differences across the intervention. B) The proposer share also increased significantly when a proposer interacted with a subject belonging to the MT group post-training (paired $t = 2.58$; $p < 0.02$) as a opposed to the CT group (paired $t = 1.6$; $p < 0.1$).

Table S1.

Conjunction analysis between MT and CT groups in the pre-training condition. 1st order linear parametric regressor that scales with unfairness separately for the MT group (n=26) and the CT group (n=24).

Region	Voxels	z-score	MNI (x y z)
L anterior insula*	341	4.86	-32 14 14
R anterior insula	19	3.50	34 10 8
R medial frontal gyrus	22	5.44	8 60 16
L medial frontal gyrus	11	5.16	-8 60 20
L dorsal ACC (BA 32)	39	3.76	-8 14 44
R dorsal ACC (BA 32)	69	3.85	10 14 44
L ventral ACC (BA 24)	46	3.82	-8 24 22
R ventral ACC (BA 24)	53	3.81	6 24 24
R superior frontal gyrus	59	4.02	24 54 0
L superior temporal sulcus	51	3.36	-44 -38 14
R precuneus	14	3.28	20 -54 24

Activations are displayed at P < 0.001, uncorrected; * FDR-corrected at P < 0.05. Extent threshold > 10 voxels. AAC, anterior cingulate cortex; BA, Brodmann Area.

Table S2.
Interaction contrast post-training [(Post CT > Pre CT) > (Post MT > Pre MT)] of the most cooperative subjects CT (n=10) and MT (n=17).

Region	Voxels	z-score	MNI (x y z)
R posterior parietal cortex	62	3.37	44 -56 26
L posterior parietal cortex	33	3.67	-40 -56 46
L midbrain	16	3.11	-4 -16 -6
R medial frontal gyrus	95	3.08	28 62 16
L superior frontal gyrus	23	3.04	-6 30 50
**L dlPFC**	**95**	**2.99**	**-48 32 22**
**R dlPFC**	**125**	**2.95**	**38 34 30**

Activations are displayed at P < 0.005, uncorrected. Extent threshold > 10 voxels. BA, Brodmann Area.
